# Polysaccharide-hydrolysing enzymes enhance the *in vitro* cleaning efficiency of Nanofiltration membranes

**DOI:** 10.3934/microbiol.2019.4.368

**Published:** 2019-12-17

**Authors:** Ahmed Houari, Patrick Di Martino

**Affiliations:** Laboratoire ERRMECe-EA1391, Université de Cergy-Pontoise, rue Descartes site de Neuville-sur-Oise 95031 Cergy-Pontoise, cedex France

**Keywords:** nanofiltration, biofouling, biofilm, cleaning, enzyme, polysaccharidase

## Abstract

The development of biofilm on the surface of filtration membranes is the main fouling component of water filtration systems. Chemical cleaning is only partially effective in removing biofilm components from the membrane surface. In order to identify opportunities to improve the efficiency of commercial cleaning solutions used in nanofiltration, we compared the *in vitro* efficacy of different commercial treatments, with or without the addition of polysaccharidases, to clean fouled membrane samples. The treatments were tested at two stages of biofilm development corresponding to 80 (D80) and 475 (D475) days of filtration in an industrial plant. The cleaning efficiency was evaluated by comparing the ATR-FTIR spectra before and after cleaning. At D80 and D475, all cleaning solutions led to a reduction of infrared signals from the biofilm. At D80, enzymatic alkaline detergent (AEDT) treatment was significantly more effective than alkaline detergent (ADT) treatment in removing proteins, but no significant difference in efficacy between the two treatments was observed for polysaccharides. The addition of polysaccharidases to AEDT did not bring any significant efficiency gain. At D475, ADT and AEDT treatments had the same efficacy, but the addition of polysaccharidases to the AEDT treatment significantly increased the removal of polysaccharides and proteins from the membrane surface. In conclusion, polysaccharidases can increase the *in vitro* efficacy of a commercially available alkaline enzymatic detergent cleaning solution against sufficiently developed biofilms. These results pave the way for the development of new cleaning solutions containing polysaccharide degrading enzymes for the cleaning of membranes used in the production of drinking water. Further experiments are needed to characterize the mechanism of this polysaccharidase effect and to confirm this increase in cleaning efficiency in an industrial context.

## 1. Introduction

Membrane fouling during filtration is the main limitation of this type of process [1],[2]. Preventive and curative treatments help to limit fouling and to maintain efficient filtration flows [3]–[5]. While inorganic fouling is generally controlled, organic fouling and in particular fouling of biological origin is not. Biofilm development on filtration membrane surfaces, also known as biofouling, is the major fouling component of water filtration systems [5]–[7]. Biofouling is a sequential phenomenon harbouring initial stages of microbial attachment to the membrane and later stages of cell multiplication and extracellular polymeric substances (EPS) production leading to biofilm development [8],[9]. The increase in size of the structure via cell multiplication and the synthesis of matrix corresponds to the stage of maturation of the biofilm. During the maturation process of biofilms formed on nanofiltration (NF) membranes, there is a diversification of the polysaccharide residues of the matrix, development of the polysaccharide network and reinforcement of the cohesion of the matrix by increase of the viscosity and the elasticity [9]. At this stage, shear forces can only tear off a fragment of biofilm when the structure becomes too prominent, limiting the growth of the biofilm in thickness and facilitating the colonization of other sites [10]. Another factor facilitating the geographic expansion of the biofilm is the active detachment of microorganisms that return to the liquid phase [11],[12]. This active detachment involves the production of microbial enzymes to degrade the matrix locally and release sessile bacteria [13].

The biofilm matrix forms a gel structure composed of EPS, mainly polysaccharides, proteins, and nucleic acids and accounts for up to 90% of the dry mass of the biofilm [14]–[17]. In a mature biofilm formed on the surface of nanofiltration membranes in a drinking water production plant, galactoside residues and β-glycan bonds are dominant in the polysaccharide part of the foulants [7]. Peanut agglutinin and wheat germ agglutinin, recognizing the motifs Galβ1-3GalNAcα1-Ser/Thr, and GlcNAcβ1-4GlcNAcβ1-4GlcNAc, respectively, bind strongly to the polysaccharides of the NF biofilm matrix. Other residues are present, and there is some variability in the proportions of the different polysaccharides in the biofilm matrix, depending on the season and the stage of maturity [7],[9]. The matrix polysaccharides are located mainly between cells and organized into entangled fibres of different lengths and cloudy zones. EPS serve as an anchoring cement and protective enclosure for attached microorganisms, rendering mechanical treatments, biocidal treatments and physicochemical treatments less effective [18]. Among the physicochemical treatments against membrane fouling, the acid treatments have a certain efficiency for the release of a part of the fixed inorganic foulants [19]. Alkaline and chelator treatments are more effective than acid treatments in restoring an increased filtration flow [4], [20]–[22]. Alkaline treatments can partially eliminate the biological fouling, fouling associated with natural organic materials and mineral substances. Chelating treatments induce, by the capture of metal ions, the blocking of inorganic, organic and even biological materials. Treatment with anionic surfactants at basic pH shows some cleaning efficiency [23], whereas treatment with cationic surfactants is inconclusive [24]. The anionic surfactant SDS has higher efficiency to remove lipids than polysaccharides and DNA from fouled nanofiltration membranes [25]. Nonionic surfactants reduce the amounts of biofilm and live microorganisms but with limited efficiency [26]. In general, chemical treatments have an interesting efficiency, although partial, but can also induce membrane alterations [27],[28].

Enzyme-containing cleaning solutions can be effective for the treatment of biofilms [29]–[32]. The advantages of the enzymatic treatments are their specificity for a target, the optimal temperatures of use generally not exceeding 50 °C, the pH of optimal use of the order of the physiological pH, a short duration of action if the enzyme concentration is optimal, the biodegradability of enzymes and their limited life in an industrial or natural environment [33]. Thus, enzymatic treatments do not degrade the filtration membranes, and limit additional costs for the treatment of waste. However, since biofilms are complex and heterogeneous, the use of a cleaning solution containing several enzymes seems necessary [7],[34],[35]. In addition, an effective cleaning protocol is usually an association of different products used simultaneously or sequentially [36],[37]. The temperature, the pH, the ionic strength, the concentration of each of the products, their time and their order of application play a key role in the optimization of cleaning processes [22]. Conventional industrial protocols for cleaning nanofiltration membranes use acidic, basic, and detergent solutions [36],[38]. However, these protocols are partially effective, particularly for the removal of biofilm matrix components [35],[39].

In order to identify possibilities for improving the efficiency of commercial cleaning solutions used in nanofiltration membrane practice, we compared the *in vitro* efficiency of different types of treatments on samples from membranes operating in a drinking water production plant. We used commercial cleaning solutions to which we added or not the two polysaccharidases lactase and pectinase. Both enzymes cleave β-glycan bonds that are widely present in NF biofilms. Lactase cleaves β-D-galactopyranosyl (1→4) β-D-glucopyranose into glucose and galactose. Pectinase contains a polygalacturonase activity and a lower proportion of cellulase activity hydrolyzing respectively the bonds between 2 galactoses in galacturonic acid and glucose polymers. The treatments were tested at two stages of formation of the biofouling deposit corresponding to different levels of maturity of the biofilm.

## 2. Materials and methods

**Figure 1.**
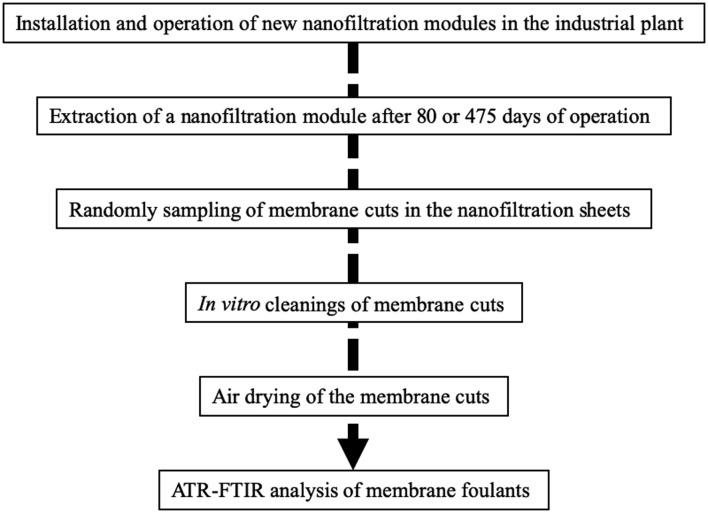
Flow chart of membrane samples preparation process.

The flow chart of membrane samples preparation process is shown in Figure 1.

### 2.1. Membrane autopsy and cleaning experiments

The filtration modules containing new NF200 B-400 membranes (DOW, La Plaine Saint Denis, France) were installed in stage 1 of the integrated pilot at the Méry-sur-Oise industrial plant and extracted after 80 and 475 operating days as previously described [9].

The *in vitro* cleanings were performed on randomly chosen membrane samples cut of 1 cm^2^ from an extracted module. Each cleaning protocol was repeated three times on three different membrane coupons. Three static bath cleaning protocols were applied (Table 1). Cleaning protocols were identical for all the steps except a step of application of a different commercial active ingredient. Three types of active ingredients were used. P3-Ultrasil^®^ 110 (Ecolab) is an alkaline detergent treatment (ADT). P3-Ultrasil^®^ 67 (Ecolab) is a neutral liquid detergent containing a combination of stabilized enzymes and surfactants. P3-Ultrasil^®^ 69 (Ecolab) is a mild alkaline liquid detergent containing a combination of organic and inorganic sequestering agents and buffers. The combination of P3-Ultrasil^®^ 67 and P3-Ultrasil^®^ 69 is an alkaline enzymatic detergent treatment (AEDT). *Aspergillus oryzae* lactase (Sigma-Aldrich, Saint Quentin Fallavier, France), and *Aspergillus niger* pectinase (Sigma-Aldrich, Saint Quentin Fallavier, France) are two polysaccharidases. Lactase and pectinase associated together to the AEDT treatment was called the multi-enzymatic treatment (MET). The percentages of active products indicated in Table 1 are in volume / volume. Ultrapure water was a bi-distilled water of 18 MΩ quality.

**Table 1.** Static bath cleaning protocols applied to biofouled NF membrane samples.

Alkaline detergent treatment (ADT)	Alkaline enzymatic detergent treatment (AEDT)	Multi-enzymatic treatment (MET)
Rinsing with ultrapure water	Rinsing with ultrapure water	Rinsing with ultrapure water
P3-Ultrasil^®^ 110 (0.5%)	P3-Ultrasil^®^ 67 (0.5%), P3-Ultrasil^®^ 69 (1%)	Lactase (1%), pectinase (1%)
Incubation 4h at 35 °C	Incubation 6h at 39 °C	Incubation 6h at 35 °C
Rinsing with ultrapure water at 30 °C
P3-Ultrasil^®^ 67 (0.5%), P3-Ultrasil^®^ 69 (1%)
Incubation 6h at 39 °C
Rinsing with ultrapure water at 30 °C	Rinsing with ultrapure water at 30 °C	Rinsing with ultrapure water at 30 °C
Citric acid (0.6%)	Citric acid (0.6%)	Citric acid (0.6%)
Incubation 4h at 30 °C	Incubation 4h at 30 °C	Incubation 4h at 30 °C
Rinsing with ultrapure water at 30 °C	Rinsing with ultrapure water at 30 °C	Rinsing with ultrapure water at 30 °C

### 2.2. ATR-FTIR analysis of membrane foulants

Sample cuts of fouled membranes were air dried for 24h at 40 °C before analysis by ATR-FTIR as previously described [40]. A Tensor 27 IR spectrophotometer with a diamond/ZeSe flat plate crystal (Bruker Optics, Marne la Vallée, France) was used to record IR spectra with air as the background and a resolution of 2 cm^−1^. Each spectrum presented is the mean of 15 spectra corresponding to different areas of the membrane surface. All the samples were pressed with the same force to obtain equivalent close contact between sample surface and ATR crystal. The membrane IR signal near 700 cm^−1^ was used to calculate ratio corresponding to the relative IR signals of proteins (band at 1650 cm^−1^/membrane signal) and polysaccharides (band at 1040 cm^−1^/membrane signal). Means ± standard deviations of the relative IR signals of proteins and polysaccharides are presented.

### 2.3. Statistical analysis

The equal-variance Student's t test, following the Fisher's test was used to determine the statistical significance of differences. *P* values below 0.05, 0.01, or 0.001 were considered significant, highly significant, or very highly significant, respectively.

## 3. Results and discussion

### 3.1. Enhanced membrane biofouling over time

**Figure 2.**
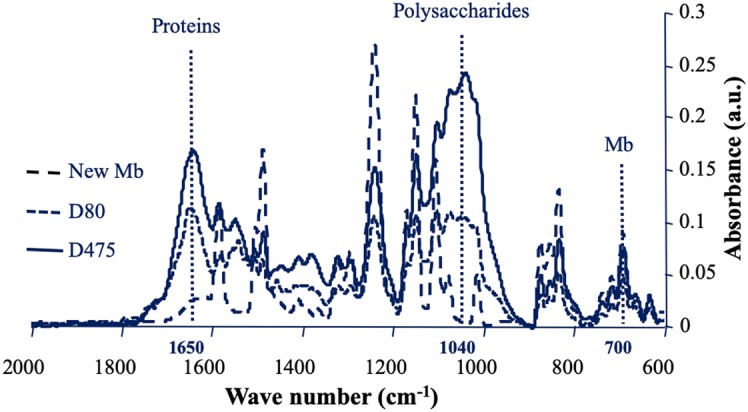
Comparison of the ATR-FTIR spectra of the new NF200 B-400 membrane and of the corresponding fouled membrane samples after 80 days (D80) and 475 days (D475) of filtration. Each spectrum presented is the mean of 15 spectra corresponding to different areas of the membrane surface. Mb: membrane.

Biofoulants present on the surface of nanofiltration membranes after different filtration times were analysed by ATR-FTIR. IR spectra of fouled membranes are presented in Figure 2 and corresponding relative IR signals of proteins and polysaccharides are presented in Table 2. As observed previously, a certain heterogeneity has been measured between different zones of the membrane, materialized by standard deviations of the relative values of proteins and polysaccharides of the fouling material, which are sometimes high [7],[9]. This emphasizes the importance of collecting fouled membrane samples in different areas and multiplying the IR spectral acquisitions at different points on the surface of each sample. After 80 days of operation (D80), the membrane IR signals were attenuated but the majority of them remained clearly visible. A large quantity of biological macromolecules was accumulated on the surface of the nanofiltration membrane (Figure 2, Table 2). Proteins, materialized by the amide I signal at 1650 cm^−1^, and polysaccharides materialized by a broad complex region of signals between 1200 and 900 cm^−1^, were found to be the main foulants as previously described [9],[41]. At D80, the region corresponding to the polysaccharides was composed of 4 peaks around 1080, 1040, 1000 and 970 cm^−1^. This reveals the diversity of polysaccharide signals at this stage of biofilm development. After 475 days of filtration (D475), the polysaccharide signals became dominant, which shows that at this stage, the biofilm matrix has developed very strongly. The peak at 1040 cm^−1^ became the major signal among the various polysaccharide signals, as for membranes in operation for several years [35]. Unlike most peaks of the membrane which are largely masked by signals of fouling material at D475, the signal close to 700 cm^−1^ remains clearly visible, which makes it possible to calculate ratios corresponding to relative IR signals of proteins (1650 cm^−1^/700 cm^−1^) and polysaccharides (1040 cm^−1^/700 cm^−1^). The means ± standard deviations of the relative IR signals corresponding to the different spectra of membrane samples are presented in Table 2. At D475, compared to D80, the relative values of the IR signals of the proteins did not change significantly, while the relative values of the polysaccharide signals increased significantly. This has already been associated with stagnation of sessile bacterial density and a joint increase of matrix polysaccharides during biofilm growth [9].

**Table 2.** Relative IR signals of membrane samples before and after cleaning.

Days of operation (Biofilm age)	Cleaning protocol	Relative IR biofilm signals	
		Proteins	Polysaccharides
D80	-	2.5 ± 0.1	2.2 ± 0.3
D80	ADT	1.3 ± 0.3***	1.0 ± 0.4***
D80	AEDT	1.0 ± 0.3***^#^	0.8 ± 0.6***
D80	MET	0.9 ± 0.2***^##^	0.6 ± 0 .4***^#^
D475	-	2.6 ± 1.3	3.9 ± 2.3^‡‡^
D475	ADT	1.6 ± 0.6***	2.7 ± 1.2**
D475	AEDT	1.5 ± 0.4***	2.7 ± 0.9**
D475	MET	1.3 ± 0.5***^††^	2.3 ± 1.2**^†^

ADT: Alkaline detergent treatment; AEDT: Alkaline enzymatic detergent treatment; MET: Multi-enzymatic treatment; Means and standard deviation of relative IR biofilm signals are presented; ***, **, *: Value differs significantly (*P* < 0.001, *P* < 0.01, and *P* < 0.05, respectively) from the value obtained before cleaning; ^‡‡^: Value differs significantly (*P* < 0.01) from the corresponding value obtained at D80; ^##^, ^#^: Value differs significantly (*P* < 0.01, and *P* < 0.05) from the value obtained after cleaning with the ADT protocol; ^††^, ^†^: Value differs significantly (*P* < 0.01, and *P* < 0.05, respectively) from the value obtained after cleaning with the AEDT protocol.

### 3.2. Cleaning experiments

Many commercially available cleaning agents can be used for nanofiltration membrane remediation [42]. The inorganic foulants of the deposit accumulated on the filtration membrane is largely eliminated by the current chemical cleanings, which is not the case of the fouling organic material and in particular deposit polysaccharides of the biofilm matrix [35]. It is therefore necessary to carry out efficacy studies of new anti-biofilm cleaning solutions to improve the efficiency of industrial cleaning. Before performing these tests on a large scale, a preliminary *in vitro* testing step on samples from membranes operating in a water production plant is a good alternative [40]. The effectiveness of three different cleaning protocols according to the use or not of alkaline detergents, surfactants, organic and inorganic sequestering agents, enzymes and in particular polysaccharidases has been evaluated *in vitro* with the membrane samples described above. The treatments consisted in commercial cleaning solutions, and polysaccharidases.

**Figure 3.**
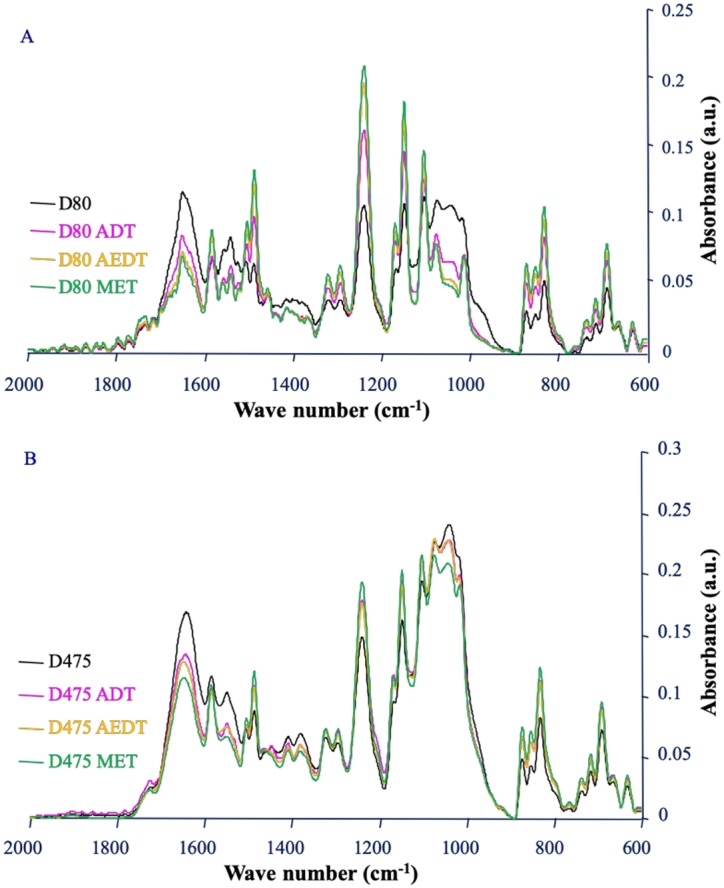
Comparison of the ATR-FTIR spectra of the fouled membrane samples before and after cleaning with the three protocols. D80 and D475: 80 and 475 days of filtration before analysis, respectively. Each spectrum presented is the mean of 15 spectra corresponding to different areas of the membrane surface. ADT: Alkaline detergent treatment; AEDT: Alkaline enzymatic detergent treatment; MET: Multi-enzymatic treatment.

After 80 days of operation, all cleaning protocols had an effect on the biofilm (Figure 3A). Whatever the type of treatment applied, the comparison of the IR spectra before and after cleaning revealed a decrease of the amide I signal and of the band corresponding to the polysaccharides. All the decreases of the foulant signals were significant (Table 2). When comparing the treatments with each other, the alkaline enzymatic detergent treatment (AEDT) was significantly more effective than the alkaline detergent treatment (ADT) in removing proteins but no significant difference in efficacy between the two treatments was observed towards the polysaccharides. The addition of polysaccharidases to AEDT (MET) provided no significant gain in efficiency at this stage of biofilm development. After 475 days of operation, significant decreases in signals of proteins and polysaccharides were also observed on the spectra corresponding to the three treatments compared to no treatment (Figure 3B, Table 2). At this stage, treatments ADT and AEDT had the same efficiency, but the addition of polysaccharidases to treatment AEDT corresponding to treatment MET significantly increased removal of polysaccharides and proteins from the membrane surface. This suggests a reduction of polysaccharides in biofilm biomass after the action of polysaccharidases. A cocktail of polysaccharide-hydrolysing enzymes has been previously shown to remove bacterial biofilm from different solid substrata in laboratory conditions [34]. Alkaline treatments destabilize the microbial membrane, denature proteins and induce the unfolding of extracellular polymeric substances [43]. On the mature biofilm formed after 475 days, the polysaccharidases have a synergistic action with the alkaline enzymatic detergent treatment. Chelating agents, surfactants and enzymes have been previously shown to act synergistically [31]. This synergistic effect could be related to a better diffusion of enzymes within the biofilm during the action of polysaccharidases since the attack of a gel structure like a biofilm by an enzyme is limited by diffusion phenomena [44]. This particular effect associated to polysaccharidases is consistent with the prevalence of polysaccharides in the matrix of NF biofilms formed after 475 days (Figure 3).

## 4. Conclusions

There is a need to enhance the efficiency of cleaning procedures to remove biofilms on the surface of nanofiltration membranes used for drinking water production. Despite their efficiency to maintain nanofiltration performance over time through flux recovery, commercial cleaning solutions are only partially efficient against the biofouling deposit. The results presented here showed that polysaccharide-hydrolysing enzymes can increase the *in vitro* efficiency of a commercially available alkaline enzymatic detergent cleaning solution. Further experiments are needed to characterize the mechanism of this polysaccharidase effect and to confirm this increase of cleaning efficiency in an industrial context.
